# Three Years with the Army of the Potomac—A Personal Military History

**Published:** 1912-08

**Authors:** William Warren Potter

**Affiliations:** Buffalo, N. Y., Brevet Lieutenant Colonel, U. S. Volunteers; Surgeon in Charge of First Division Field Hospital Second Army Corps; Surgeon 57th Regiment New York Volunteers; Assistant Surgeon 49th Regiment New York Volunteers; Recorder Second Division Hospital Sixth Army Corps, etc., etc.


### Three Years with the Army of the Potomac—A Personal Military History

By William Warren Potter, M.D., Buffalo, N. Y., Brevet Lieutenant Colonel, U. S. Volunteers; Surgeon in Charge of First Division Field Hospital Second Army Corps; Surgeon 57th Regiment New York Volunteers; Assistant Surgeon 49th Regiment New York Volunteers; Recorder Second Division Hospital Sixth Army Corps, etc., etc.

*2d. Spottsylvania.*

We have been almost constantly fighting for the past seven days, and are now confronting the enemy with a prospect of renewing the contest almost any moment, our lines being near Spottsylvania C. H. General Sedgwick, the Commander of the 6th Corps, was killed on the 9th near Todd's Tavern, by a sharpshooter while he was reconnoitering the enemy's position from a redoubt. I saw Colonel McMahon, of Sedgwick's staff, very soon afterwards, and he told me the General was wounded in the head and fell backwards into his (McMahon's) arms, expiring almost instantly. The 57th regiment has been sent to Fredericksburg to do provost guard duty for the present.

May 13. Friday. Still at Cossin's. Yesterday morning, May 12, at four o'clock the Second Corps, after marching all night from our position on the right, attacked the enemy near his center, and went over the works with a rush, the First Division in the lead in two columns of masses, each regiment doubled on the center, General Barlow leading the charge in his shirt sleeves. We had forty-five cannon in our possession at one time during the charge, and brought off twenty-two of them, capturing also 4,000 prisoners, including Generals Ed. Johnson and George H. Stuart, besides some colors. Our loss was heavy, and I had 1200 wounded in my hosiptal before noon. I have 950 in the hospital to-day, after sending 450 to Fredericksburg yesterday. Colonel F. A. Walker was injured by a fall from his horse on the 10th, and is now in my tent. General Barlow has sent the Division band to play for the wounded in hospital, which is the first music we have been allowed since crossing the Rapidan.

May 15. Sunday. I worked all night last night shipping the wounded to Fredericksburg, using 250 army wagons, all the ambulances and spring wagons of the Division, fifty in number, and besides sent a large number on foot. After all the transportation was exhausted, about 200 were still unprovided for, and these I was ordered to leave behind, as the Corps was moving to the left thus uncovering the hospital; so it must be broken up.

Nurses and rations were provided and everything possible was done for their comfort. I was the last authorized person to leave, which I did about eight o'clock A. M., and within fifteen minutes after Rosser came in with a light brigade, capturing the place, paroling the men, and taking the extra rations for his hungry horde. We are not fighting to-day, but shall probably resume business in a day or two. Lieutenant Colonel Strycker, 2d Del., was killed on the 12th, and Colonel Frank has been relieved of the command of the Third Brigade, reason not known to me. The charge of the Second Corps on the 12th is regarded as one of the most brilliant and effective of the war.

May 17. Tuesday. The 49th has suffered severely in the recent battles, ten officers being reported killed and six wounded; Colonel Bidwell is safe. I saw Dr. Hall yesterday, who gave me these particulars. I notice all the papers speak of General Grant as though he commanded the Army of the Potomac. General Meade is in immediate command and directs the movements, but, of course, under Grant's advice and guidance as to the general plan.

May 19. Yesterday morning we had a fight, losing about 500 men, Lieutenant Hall, 57th, on Colonel Frank's staff, among the number. I have had no rest for two nights past, from the fact that we have moved our hospital during both nights. The papers speak of the flying enemy, conveying the idea that they are going so fast that we can hardly overtake the routed columns. The truth is we are not ten miles from Fredericksburg yet, and the 2d Corps has not yet passed Spottsylvania C. H. We have sent all our wounded to the rear, and are under orders to move at two o'clock A. M. tomorrow, prepared for a long march. Dr. Houston is on my right writing a letter home. He keeps up good spirits and is in excellent health, enduring the fatigue well for a man of his years. General Grant's Headquarters are very near us just now, and I saw him this P. M. The enemy is strongly fortified on this line, and it is doubtful if we can drive him out, though there is a rumor today that he is falling back towards Richmond.

May 20. Our mails are very irregular and infrequent, though to-day I received home papers to the 12th. Miss Hancock is at Fredericksburg attending to the wounded of the First Division, and is reported as performing excellent service. Last night two Brigades of Ewell's corps made an attack upon our communications with Fredericksburg, but was handsomely repulsed by the new troops of Tyler's Division from the defences of Washington. Today it is very warm and a move seems on the tapis.

*3d. The North Anna.*

May 24. Tuesday. For the past three or four days and nights we have been almost constantly on the move. On the night of the 20th, at eleven o'clock, the corps started for the Mattapony River, by a detour around the right flank of the enemy, and in the early morning of the 21st we passed through Bowling Green, a beautiful little village hitherto untouched by the hand of war. By ten o'clock A. M. we had reached Milford Station, and soon established ourselves about a mile over the Mattapony, where we threw up entrenchments and awaited the enemy who came not. We held the position until the morning of the 23d, having meanwhile been joined by the rest of the army, when we set out for the North Anna, arriving at Chesterfield the same afternoon, and here we are. We are about four miles from Hanover Junction, where we heard the rebel cars whistle when we came up yesterday. We had quite a sharp fight last night, the loss chiefly occurring in Birney's Division. Skirmishing and some cannonading are going on to-day, but no general engagement. It is again rumored that the rebels are falling farther back, but I do not credit it.

Thursday, May 26. Still at the North Anna, and it is hot. The Division is across the river holding an intrenched line, while the hospital is holding a comfortable farm house on the north bank unintrenched. I sent a pail of lemonade over to General Hancock and staff, and one to General Barlow and his staff as well, to cool their parched throats this hot afternoon. Major Ellis was wounded on the 12th by a ramrod in the arm, but it is not reported as dangerous. Captain Arnold lost one of his guns at the Po River on the 10th—the first the 2d corps ever lost—through no fault of his- however. The gun became entangled in a small tree when the Division retired, and, as it could not be extracted readily, it was disabled and left to comfort the enemy. It is reported that Lee will make a big stand here—just the contrary of the rumor yesterday, as will be observed; but we shall see which is correct. The 57th is still at Fredericksburg doing provost duty, Captain Wright acting as Provost-Marshal.

*The Totopotomoy.*

May 30. Monday. We have crossed the Pamunkey River and are again on the Peninsula, our front line of battle being, it is stated, not more than twelve miles from Richmond. We left the North Anna on the 27th, bivouacking that night within three miles of the Pamunkey. The 28th and 29th were spent in reconnoitering and skirmishing, and today we are confronting a line of the enemey's works at the Totopotomoy.

May 31. Still at it on the Totopotomoy line. It is a very hot day, and a ration of whiskey has just been issued to the troops. About two o'clock P. M. Captain Rose, C. S. Third Brigade, came to my quarters in a greatly "demoralized" state, and soon fell fast asleep on the ground inside the tent. At nightfall we were ordered to move, and after loading the wounded into the ambulances and packing up everything, I put Rose into the leading ambulance alone, when we started for Cold Harbor. During the night a wounded man crept in alongside of Rose, and when he awoke in the gray of the morning he—Rose—thought he had been wounded also, and began a self examination with a view to determine as to the fact. He told of this himself afterwards, to the great amusement of all his friends. Colonel Brown, 145th Pa., has not been heard from or of since the 12th, and it is supposed he was captured at the assault at the salient. I saw his dog "Spot" today at the hospital, looking as though he had lost his last and only friend. I hear Captain R. B. Heacock, of the 49th, was killed at Spottsylvania on the 18th. Dr. George L Potter and I made an operation yesterday upon Lieutenant Hunt of Arnold's battery. He was struck in the foot with a piece of shell about noon, and we removed all the fractured bones, hoping to save the foot. (Lieutenant Hunt died in Washington a few days afterward, with lockjaw.)

*4th Cold Water Harbor.*

June 2. Thursday. Today is hot and dusty. The Second Corps is now the right of the Army, for the first time since we started out, the Sixth Corps having been moved from the right toward the left in the night. Yesterday the First Division had two hundred wounded, and all were sent away but thirty-five. Thus far today (twelve M.) there has been no fighting, only an occasional gun being heard along the lines. We heard a rumor a short time ago that the enemy was preparing to attack our right flank, and that we might be compelled, if he did, to move our hospital to keep it out of danger of probable capture. An officer from General Meade's headquarters just told me that General "Baldy" Smith had joined the Army of the Potomac on the left, with 20,000 men. Hurrah! Our depot is now at the White House, and our position is very near where we were two years ago under McClellan.

Friday, June 3. We had a severe battle this morning in which the First Division lost heavily. I cannot say how many now, but I have already received over five hundred in hospital, and the wounded are being brought in constantly. We are but a short distance from Cold Harbor, yet it seems very difficult to get quite there. The Nemesis of the Army of the Potomac appears to be enshrined in that region. Colonel J. C. Drake, 112th New York, formerly Captain in the 49th, was killed day before yesterday. I saw Dr. Hall this morning, and he is well. Colonel Morris, 66th New York, Colonel Peter A. Porter, 8 New York Artillery, and Colonel McKeon, 81st Pennsylvania, were killed this morning, all commanding Brigades. Their bodies were brought to my hospital, whence they were sent north. The 57th had not returned from Fredericksburg yet, though I presume it will join us soon. I have shipped 423 wounded to the White House today.

June 4. Sent another consignment of wounded to the White House today, numbering 255, and shall probably send more tomorrow. General Grant's Headquarters are within a quarter of a mile of my hospital, and I see him quite often. No fighting today excepting skirmishing, and an occasional gun. The 57th has returned; Captain Wright and other officers are looking well, but they will be obliged to take part in the heavy fighting now, and I fear the result to the remnant of that once splendid regiment. Dr. Gesner is also back again, but he is given to complaining so much that he is no longer an interesting companion. S. B. Butler ("Brain") called on me yesterday and, as he was not feeling well, I gave him a little Sp. Frumenti, which seemed too brace him up.

Monday, June 6. I sent away 357 more wounded yesterday, making 1035 in the last three days. Today we moved the hospital about two miles towards the left, and are now at the Tyler House, on the road leading from Cold Harbor to the White House. This estate was formerly owned by Dr. Tyler, a cousin of Ex-president Tyler, who died while we were in this vicinity two years ago. We are about two miles from Dr. Gaines' farm, where we camped in May, 1862, and where Porter fought his great battle on the 27th of June, 1862. We are not fighting very heavily these days, but receive about one hundred wounded daily into my hospital. We occupy the house for office and mess purposes, and our tents are pitched on the lawn in front. Colonel Brooke commanding the Fourth Brigade was wounded two days ago, and Colonel Beaver now commands that Brigade. Information has been received that Colonel Brown is in Richmond and unhurt. It is thundering heavily, and a rainstorm is imminent. The 57th is in the front line of breastworks, doing duty at the post of danger in its accustomed good way. Captain McCuen, Provost-Marshal at Corps Headquarters, had one of his legs shot off last evening in front of General Hancock's tent. Hancock is considered by all as the hight hand corps commander of this Army, one who is always where he is wanted at the critical moment, and who punishes the enemy severely when he goes monkeying around the Second Corps. In many respects he is an ideal field commander, and it would be difficult to supply his place, should anything happen to render this necessary.

June 8. Still at the Tyler House. Mrs. General Barlow and Miss Hancock arrived yesterday morning, and are my guests, Miss Hancock busying herself in caring for the wounded. We have a large ice house on the premises filled with native ice, which is a great comfort to the wounded—and the unwounded. General Barlow has just been here to see his wife, and found her supplying the wounded with milk punch, while we are loading them for shipment to White House.

June 9. Thursday. I have just had orders to send all my wounded to the rear at White House, which looks like a move tonight. Dr. John S. Billings, Assistant Surgeon U. S. A., Medical Inspector A. of P., visits the hospital every day, spending some time in unofficial intercourse with us, as he finds it a convenient place for obtaining a little rest when he needs it. He has been a frequent guest at odd times during the campaign, and seems to enjoy himself here very much. We always try to make him feel at home, and quite welcome whether for duty or otherwise. Mrs. Barlow and Miss Hancock are with us yet, and may remain for some time. Captain Arnold's time has expired, so he has been mustered out, and he went home day before yesterday. I met Captain Elliot on the 7th; he was well and appeared in good spirits. I should not be surprised if we turned up on the James River next.

Saturday, June 11. The news of the sudden death of James Prince at the Mansion House in Buffalo, has just reached me. It was a great shock, as he always appeared the picture of perfect health. Thurlow Weed was at the hospital today, and expressed himself as greatly pleased with the care we give the wounded in our field hospitals, and particularly this one. I am sending away my wounded again today, and Mrs. Barlow and Miss Hancock will return to the White House with this lot. From present appearances I think we shall move tonight.

Sunday, June 12. This is one of the most delightful days we have had since the campaign opened—an ideal summer day in every respect. Religious services were held on the lawn in front of the house this morning, the First Division Band furnishing the music, playing, among other airs, the Doxology at the close. It was an impressive and interesting scene to see the soldiers that could get out of the tents gather around, and enter into the spirit of the service, as much as though it were in the finest church. Dr. Aiken expects to start home tomorrow, as his regiment's time expired today. I am sending away more wounded today, and Dr. Dougherty informs me that we shall positively move from here tonight. I suppose there is great anxiety at the North just now as to our operations; but patience, patience. We have more men than McClellan had on the Peninsula, and the rebels are weaker; yet we need re-inforcements, and Grant will have them. Neither Stanton nor Halleck can prevent it this time. Stanton's principal occupation seems to be, just now, to telegraph bulletins to General Dix at Fort Monroe.

*5th Petersburg.*

June 18. Saturday. We have crossed the Chickahominy and James Rivers, and are now fighting a great battle before Petersburg. I have already 850 in my hospital, and the cannonading is terrific. The 57th is nearly wiped out, all the officers but two being wounded—some of them mortally. Captain Wright is wounded in the foot; (afterwards amputated and died ; Captain Alcoke in the chest; (he lost an arm at Fredericksburg) ; Major Kirke, mortally; Captain Middleton, slightly; Adjutant Case, flesh wound in the neck; Lieutenant Brower and Britton both have flesh wounds and will recover. The regiment had but forty-five men for duty yesterday. This is the third day of the battle, and the end is not yet. Mrs. General Barlow is here, with some members of the Sanitary Commission, dispensing luxuries to the wounded. Colonel Beaver is slightly wounded in the side, and will go home to recover. The little drummer boy of the 61st, George Nims, has received a most frightful wound, his face having been nearly torn off by a shell last night.

We left the Tyler House at dark Sunday night, marched all night the 12th, all day the 13th, resting at night; marched all day the 14th and 15th until daylight the 16th, when we reached the James River. The troops had already crossed the day before, but as I did not feel well I remained with the main hospital and ambulance train, crossing the James with it about three o'clock P. M. of the 16th, on a pontoon bridge between Wilcox Landing and Windmill Point. We pushed on for Petersburg, distant sixteen or seventeen miles, where the troops were already engaged, reaching there about ten o'clock P. M., when we immediately set about putting up the hospital and gathering in the wounded, working all night at this business.

(*To Be Continued.*)

